# Supplementary material for: Patterns of Intron Gain and Loss in Fungi
Source: PLoS Biol. 2004 Nov 30;2(12):e422. doi: 10.1371/journal.pbio.0020422 (PMC532390; doi:10.1371/journal.pbio.0020422)
Supplement: Table S1 — Also available at http://genes.mit.edu/NielsenEtAl/. (4.3 MB ZIP). [file pbio.0020422.st001.zip › NielsenEtAl/html/620.html]

AN9403.1.NCU03004.1.MG10569.1.FG02782.1


```
 CLUSTAL W (1.82) Multiple Sequence Alignments - Introns Inserted


Sequence 1: NCU03004.1	379 aa
Sequence 2: MG10569.1	383 aa
Sequence 3: FG02782.1	386 aa
Sequence 4: AN9403.1	364 aa
Alignment Length: 389 aa
Number Identitical Residues: 245 aa
Alignment Score (without introns) 10557


MG10569.1 	MS--RYLRPAVRMAATAARAPALRSSTGTSSLIGSQRAA---AFRPAVSMQQRWASSGTK
NCU03004.1	MS--RYLRPAVRLAATASRVQVARP--AAPSFFKPASVA---AFQKRTYAD---APAGTK
FG02782.1 	MS--RILRPAARLAASTRAIRAPVAPSVAQSAISRAALARPVVFGSAQTRSYADNSSGVK
AN9403.1  	MASLRLFRPAARLLS--SRLSATRP-TFPQTACTPSILR----FRGYATEN------GTK
          	*:: * :***.*: :      .  . : . :            *      .      *.*

MG10569.1 	EYTVREALNEALVEELEANDKVFVMGEEVAQYNGA~YKVTKGLLDRFGERRIIDTPITEM
NCU03004.1	DYTVRDALNEALAEELEANDKVFVMGEEVAQYNGA~YKVTKGLLDRFGDRRVIDTPITEM
FG02782.1 	EYTVRDALNEALAEELDQNEKVFILGEEVAQYNGA~YKVTKGLLDRFGDKRVIDTPITES
AN9403.1  	EVTVRDALNEALAEELERNQKTFILGEEVAQYNGA2YKVTRGLLDRFGPKRVIDTPITEA
          	: ***:******.***: *:*.*::********** ****:******* :*:******* 

MG10569.1 	GFTGLAVGAALSGLHPV0CEFMTYNFAMQSIDHIVNSAAKTLYMSGGIQPCNITFRGPNG
NCU03004.1	GFTGLAVGAALSGLHPV0CEFMTFNFAMQSIDHIVNSAAKTLYMSGGIQPCNITFRGPNG
FG02782.1 	GFCGLAVGAALSGLHPV0CEFMTFNFAMQAIDQVINSAAKTLYMSGGIQPCNITFRGPNG
AN9403.1  	GFCGLAVGAALAGLHPI0-----------AIDQIINSAAKTHYMSGGIQPCNITFRGPNG
          	** ********:****:            :**:::****** ******************

MG10569.1 	FASGVGAQHSQDYSAWYGSIPGLKVVSPWSAEDAKGLLKAAIRDPNPVVVLENE~LMYGQ
NCU03004.1	FAAGVAAQHSQDYSAWYGSVPGLKVVSPWSAEDAKGLLKAAIRDPNPVIVLENE~LMYGQ
FG02782.1 	FAAGVAAQHSQDYSAWYGSIPGLKVVSPWSAEDAKGLLKAAIRDPNPVVVLENE~LMYGQ
AN9403.1  	FAAGVAAQHSQDYSAWYGSIPGLKVVAPWSAEDAKGLMKAAIRDPNPVVVLENE2LLYGQ
          	**:**.*************:******:**********:**********:***** *:***

MG10569.1 	SFPMSEAAQKDDFVIPFGKAKIERQGKDLTIVTLSRCVGQSLVAAENLKKKYGVEVEVIN
NCU03004.1	VFPMSEAAQKDDFVIPFGKAKIERAGSDLTIVTMSRCVGQSIVAAEALKKKYGVEVEVLN
FG02782.1 	SFPMSEAAQKDDFVIPFGKAKIERSGKDLTIVSLSRTVGQSLIAAENLKKKYGVEAEVIN
AN9403.1  	AFPMSEAAQKDDFVLPIGKAKIERPGKDLTIVSLSRCVGQSLNAAAELKQKYGVEAEVIN
          	 *************:*:******* *.*****::** ****: **  **:*****.**:*

MG10569.1 	LRSIKPLDINAIVQSVKKTHRLMSVESGFPAFGVGSEILALTMEYAFDYLDAPAQRVTGA
NCU03004.1	LRSIKPLDLDAILKSIKKTHRLMTVESGFPSYGVGAEIVALAVEYGFDFLDAPPQRVTGA
FG02782.1 	LRSIKPLDVETIIQSVKKTHRLLSVESGYPAFGVGSEILALTMEYGFDYLDAPAARVTGA
AN9403.1  	LRSVKPLDVETIIQSLKKTGRLMCVESGFPMFGVSSEILALSMEYGFDYLTAPAVRVTGA
          	***:****:::*::*:*** **: ****:* :**.:**:**::**.**:* **. *****

MG10569.1 	DVPTPYAQKLEEMSFPTEQIIEEHAAKMLKL-
NCU03004.1	DVPTPYAQGLEEMAFPTESLIENHAAKLLKLI
FG02782.1 	EVPTPYAQKLEEMSFPTEKLIEDYAAKVLRL-
AN9403.1  	EVPTPYAVGLETMSFPQEDTIVGQAAKLLRL-
          	:******  ** *:** *. *   ***:*:*
```
